# MEK inhibitor cobimetinib increases calreticulin and induces immune modulation in TNBC

**DOI:** 10.1007/s00109-026-02684-8

**Published:** 2026-05-25

**Authors:** Ling-Ming Tseng, Ka-Yi Lau, Ji-Lin Chen, Pei-Yi Chu, Chun-Teng Huang, Wan-Lun Wang, Yuan-Ya Chang, Jiun-I Lai, Chi-Cheng Huang, Ming-Shen Dai, Chun-Yu Liu

**Affiliations:** 1 Division of General Surgery, Department of Surgery, Taipei Veterans General Hospital, Taipei, Taiwan; 2 Comprehensive Breast Health Center, Taipei Veterans General Hospital, Taipei, Taiwan; 3 School of Medicine, College of Medicine, National Yang Ming Chiao Tung University, No. 155, Sec. 2, Li-Nong Street, Beitou District Taipei, 112304 Taiwan; 4 Division of Medical Oncology, Department of Oncology, Taipei Veterans General Hospital, Taipei, Taiwan; 5 Department of Pathology, Show Chwan Memorial Hospital, Changhua, Taiwan; 6 School of Medicine, Fu Jen Catholic University, New Taipei , Taiwan; 7 Department of Health Food, Chung Chou University of Science and Technology, Changhua, Taiwan; 8 Division of Hematology & Oncology, Department of Medicine, Yang-Ming Branch of Taipei City Hospital, Taipei, Taiwan; 9 School of Medicine, Institute of Clinical Medicine, National Yang Ming Chiao Tung University, Taipei, Taiwan; 10 Institute of Epidemiology and Preventive Medicine, College of Public Health, National Taiwan University, Taipei, Taiwan; 11 Hematology/Oncology, Tri-Service General Hospital, National Defense Medical Centre, Taipei, Taiwan

**Keywords:** Triple-negative breast cancer, Immunogenic cell death, MEK inhibitor

## Abstract

Immunogenic cell death (ICD) can activate immune cells and potentiate the response to immunotherapy. The mitogen-activated protein kinase (MAPK) signaling pathway regulates the immune response and mediates T-cell receptor signaling. Activation of the MAPK signaling pathway decreases the levels of tumor-infiltrating lymphocytes in triple-negative breast cancer (TNBC) and is associated with worse survival. Here, we investigated the potential of the MEK inhibitor cobimetinib as an ICD inducer in TNBC. We demonstrated that cobimetinib elicited hallmarks of ICD, including elevated levels of damage-associated molecular patterns. Ectopic ERK expression attenuated cobimetinib-induced CRT. Cobimetinib also evoked caspase-8 activation with increased Bax and Bak expression, while caspase inhibitor suppressed cobimetinib-induced CRT expression and apoptosis. In immunocompetent (BALB/c) mice bearing 4T1 tumors, cobimetinib exhibited stronger tumor growth inhibition than in immunodeficient (nude) mice, suggesting an immune-dependent antitumor effect. Furthermore, cobimetinib treatment increased the proportions of naïve CD8^+^ T cells and effector CD4^+^ T cells and decreases the proportion of myeloid-derived suppressor cells in immunocompetent mice bearing 4T1 tumors. Notably, we observed differential immunomodulatory effects of cobimetinib in tumor-bearing mice compared to non–tumor-bearing immunocompetent mice. Our findings indicate that MEK inhibition is associated with ICD-related molecular alterations, and that cobimetinib may contribute to immune modulation in TNBC.

**Supplementary Information:**

The online version contains supplementary material available at 10.1007/s00109-026-02684-8.

## Introduction

The success of immune checkpoint inhibitor (ICI) therapy has been established for several typically immunogenic tumors, including melanoma and non-small cell lung cancers [1]. Recent studies have indicated that triple-negative breast cancer (TNBC), a challenging subtype of breast cancer to treat, can be immunogenic compared to non-TNBC subtypes [2, 3]. Studies indicate the impact of the immune system on prognosis and chemotherapy response in TNBC. The presence of tumor-infiltrating lymphocytes (TILs) has been linked to favorable outcomes and is recognized as a valuable biomarker for assessing the response to immunotherapy [4, 5]. Early trials of ICI monotherapy in patients with metastatic TNBC have shown modest response rates. Data from phase 3 clinical trials of ICIs, including atezolizumab (an anti-programmed cell death-ligand 1 (PD-L1) monoclonal antibody) or pembrolizumab (an anti-programmed cell death-1 (PD-1) monoclonal antibody), in combination with chemotherapy have shown that TNBC tumors expressing high levels of PD-L1 (on immune cells or tumor cells) can demonstrate immunogenicity and respond to immunotherapy [1, 6, 7].

Emerging evidence suggests that conventional chemotherapy can trigger an antitumor immune response by inducing immunogenic cell death (ICD) [8]. This type of cell death is accompanied by the release of damage-associated molecular patterns (DAMPs) from stressed and dying cells. These include the exposure of calreticulin (CRT) on the cell surface, secretion of adenosine triphosphate (ATP), and the release of nuclear high mobility group box 1 (HMGB1) [9, 10]. DAMPs play a role in recruiting dendritic cells (DCs) and stimulating their maturation. Mature DCs then present antigen to CD8^+^ cytotoxic T lymphocytes and prime them against chemotherapy-resistant, residual cancer cells [11]. Combining immunogenic agents such as cyclophosphamide and 5-fluorouracil with other therapies can render cancer cells immunogenic, and cells that succumb to ICD show durable anticancer immunity [12, 13].

Mitogen-activated protein kinase (MAPK) cascades can regulate the immune response by producing immunomodulatory cytokines, such as TNFα, IL-1, and IL-10 [14, 15]. The Ras/Raf/MEK/ERK signaling pathway is an important protein kinase cascade in MAPK. Chemotherapeutics targeting MAPK cascades, such as BRAF inhibitors and MEK inhibitors, can improve the efficacy of immunotherapy [16]. Moreover, high expression levels of ERK have been shown to correlate with shorter survival in patients with TNBC [17]. Therefore, MEK/ERK are potential targets for antitumor treatments including cancer immunotherapy. Previous research has demonstrated that the activation of MAPK cascades can lead to a decrease in the levels of TILs in TNBC, a factor correlated with poorer survival rates [18]. The combination of a MEK inhibitor (trametinib or selumetinib) with ICIs exhibited a synergistic effect in TNBC mouse models [18]. Cobimetinib, a potent and highly selective small molecule MEK inhibitor approved for the treatment of advanced melanoma, has also undergone testing in several clinical trials, including those focused on breast cancer [19]. The COLET study demonstrated an increasing trend in the overall response rate in patients with PD-L1-positive disease when treated with a combination of an anti-PD-L1 antibody, paclitaxel, and cobimetinib [20]. Here, we demonstrate that cobimetinib can induce ICD and exert immunomodulatory effects in TNBC.

## Materials and methods

### Cell culture and reagents

Human TNBC cell lines (MDA-MB-231 and MDA-MB-468) and a murine TNBC cell line (4T1) were obtained from the American Type Culture Collection (Manassas, VA, USA). These cell lines were cultured in Dulbecco’s Modified Eagle’s Medium or RPMI-1640 Medium supplemented with 10% heat-inactivated fetal bovine serum (FBS), 0.1 mM non-essential amino acids, 2 mM L-glutamine, 100 U/mL penicillin G, and streptomycin sulfate (Gibco, Waltham, MA, USA) at 37 °C in a 5% CO_2_ incubator. Cobimetinib (GDC-0973, commercial name Cotellic; AdooQ, Irvine, CA, USA) was dissolved in dimethyl sulfoxide (DMSO; Sigma Aldrich, St. Louis, MO, USA) for in vitro experiments. For in vivo animal studies, cobimetinib was dissolved in a stable solvent system comprising 5% DMSO, 30% polyethylene glycol 300 (Sigma-Aldrich), 5% Tween 80 (Sigma-Aldrich), and 60% ddH_2_O.

### Transfection and small interfering RNA treatment

The plasmid pFLAG-CMV-hErk1 was obtained from Addgene (Cambridge, MA, USA). Cells were transiently transfected with the plasmid in the presence of serum-free medium using PolyJet In Vitro DNA Transfection Reagent (SignaGen laboratories, Ijamsville, MD, USA) following the manufacturer’s protocol. For siRNA transfections, siRNAs targeting human MAPK3 (L-003592), human MAPK1 (L-003555), or a control (D-001810) were transfected using DharmaFECT Transfection Reagents (Horizon Discovery, Cambridge, England) as per the manufacturer’s protocol.

### Cell viability and ATP assay

To examine cell viability, cells were seeded in 96-well plates at the following densities: 1.5 × 10^3^ cells per well for 4T1, 3 × 10^3^ cells per well for MDA-MB-231, and 4 × 10^3^ cells per well for MDA-MB-468. After incubating for 24 h, the cells were treated with or without drugs for 72 h. Subsequently, 10 µL of a 5 mg/mL MTT solution was added to each well, followed by a 3-hour incubation period. The samples were dissolved by adding 100 µL of DMSO, and the absorbance was measured at 570 nm using a microplate reader (TECAN Sunrise). To determine extracellular ATP levels, cells were plated in 24-well dishes with 250 µL of medium and incubated overnight. Subsequently, they were treated with cobimetinib at the specified concentration for 18 h, and the supernatants were evaluated using the luciferin-based CellTiter-Glo Luminescent Cell Viability Assay Kit (G7573, Promega, Madison, WI, USA).

### Flow cytometry analysis

For apoptosis detection, apoptotic cells were evaluated using an APC Annexin V (BD Biosciences, Bedford, MA, USA) and propidium iodide (Sigma-Aldrich) double staining assay. Cells were seeded in 6-well plates overnight and treated with cobimetinib for 48 h at the indicated concentration in complete medium. The treated cells were collected and suspended at a density of 2 × 10^5^ cells in 0.1 mL 1X Binding Buffer (BD Biosciences) containing APC Annexin V and propidium iodide. After a 15-minute incubation at 24 °C in the dark, the cells were analyzed using a BD LSR II flow cytometer (BD Biosciences). For detection of CRT exposure, cells were seeded in 6-well plates at the following densities: 1.5 × 10^4^ cells for 4T1, 3 × 10^4^ cells for MDA-MB-231, and 4 × 10^4^ cells for MDA-MB-468, and were treated with cobimetinib for 18 h at the indicated concentration in complete medium. After cobimetinib treatment, the cells were washed twice with 1X phosphate-buffered saline (PBS) and fixed with 0.25% paraformaldehyde in 1X PBS for 5 min. The cells were washed twice with cold 1X PBS and then incubated for 30 min in the dark with Alexa Fluor 647-conjugated anti-CRT antibody (1:100; ab196159, Abcam, Cambridge, UK) or Rabbit IgG (1:100; ab199093, Abcam), both diluted in blocking buffer (Abcam). Cell-surface CRT was then examined using a BD LSR II flow cytometer.

### Western blot analysis

As previously described [21], cell lysates were prepared using RIPA Lysis and Extraction Buffer (Thermo Fisher Scientific, Waltham, MA, USA). For the preparation of cell-membrane and cytoplasmic lysates, the Mem-PER Plus Membrane Protein Extraction Kit was employed after harvesting and washing the cells with 1X PBS. In Western blot analysis, proteins were separated by SDS-PAGE and subsequently transferred onto polyvinylidene fluoride (PVDF) membranes. Primary antibodies included anti-phospho-ERK1/2 (Thr202/Tyr204; 9101 S), anti-ERK1/2 (9102 S), anti-phospho-MEK1/2 (Ser217/221; #9154), anti-MEK1/2 (#9126S), anti-PARP (#9542S), anti-CRT (#12238S), anti-Bak (#3814S), anti-Bax (#2772S), anti-Caspase-3 (#9662S), anti-Cleaved Caspase-3 (#9661S), and anti-GAPDH (#5174) antibodies from Cell Signaling Technology (Danvers, MA, USA). The anti-Caspase-8 (NB10056116) antibody was acquired from Novus Biologicals (Centennial, CO, USA). Following washes, PVDF membranes were exposed to HRP-conjugated secondary antibodies (Millipore) for 1 h at 24 °C. Subsequently, the membranes were developed by employing Immobilon Western chemiluminescence HRP substrates from Millipore for enhanced visualization.

### Xenograft tumor growth

Female nude mice and BALB/c mice (aged four to six weeks, National Laboratory Animal Center, Taipei, Taiwan) were used. All experimental procedures involving these mice were conducted in accordance with protocols approved by the Institutional Animal Care and Use Committee of Taipei Veterans General Hospital. Each mouse received orthotopic inoculation with 2 × 10^5^ 4T1 cells suspended in 0.1 mL PBS containing 50% Matrigel (BD Biosciences) into the mammary fat pad under isoflurane anesthesia. Tumor sizes were monitored using calipers, and their volumes were calculated using the standard formula: width^2^ × length × 0.52. Once tumors reached a volume of 200 mm^3^, the mice were randomly divided into groups and administered cobimetinib orally at a dose of 10 mg/kg once daily. Tumor sizes and body weights of cobimetinib-treated and vehicle-treated mice were measured. On day 22 after treatment initiation, the mice were euthanized, and xenografted tumors were harvested for molecular analysis through Western blotting and immunohistochemical staining. For mice without tumors, they were randomly assigned to groups and orally administered cobimetinib at a dose of 10 mg/kg once daily or a vehicle. Body weights of cobimetinib-treated and vehicle-treated mice were monitored, and on day 22 after administration, the mice were euthanized and further evaluated.

### Isolation of splenocytes

Splenocytes were harvested following the previously described protocol [22]. A total of 1 × 10^6^ cells were dispensed into each tube for subsequent antibody-fluorochrome staining and analysis, following the manufacturer’s protocol. Antibodies used included Fixable Viability Stain 780, Phycoerythrin (PE)-Cy7-conjugated anti-mouse CD3, Allophycocyanin (APC)-conjugated anti-mouse CD4, Fluorescein Isothiocyanate (FITC)-conjugated anti-mouse CD8, (Brilliant Blue) BB515-conjugated anti-mouse CD11b, APC-conjugated anti-mouse CD11c, BB515-conjugated anti-mouse CD25, PE-conjugated anti-mouse CD62L, PE-conjugated anti-mouse CD80, PerCP/Cy5.5-conjugated anti-mouse CD45, PE-Cy7-conjugated anti-mouse CD86, APC-conjugated anti-mouse Gr-1, PE-conjugated anti-mouse I-A/I-E, PE-conjugated anti-mouse Foxp3 antibodies, and their corresponding IgG isotype controls, which were all purchased from BD Biosciences. PE594-conjugated anti-mouse CD44 antibody and its IgG isotype control were purchased from Biolegend (San Diego, CA, USA).

### Immunohistochemical staining

The procedure of immunohistochemical staining was performed as previously described [23]. Primary antibodies used included HMGB1 (#6893, 1:1600 dilution, Cell Signaling Technology), phospho-Erk1/2 (Thr202/Tyr204, #4370, 1:200 dilution, Cell Signaling Technology), Erk1/2 (#ab17942, 1:400 dilution, Abcam), CRT (ab2907, 1:500 dilution, Abcam), CK18/M30 (No. 10700, 1:300 dilution, PEVIVA), CD4 (#25229, 1:50 dilution, Cell Signaling Technology), and CD8α (#98941, 1:200 dilution, Cell Signaling Technology).

### Immunofluorescence staining

Immunofluorescence staining was performed on formalin-fixed paraffin-embedded 4T1 tumor sections as previously described [22]. Briefly, sections were incubated with primary antibodies against CD4 (1:20; Thermo Fisher Scientific), Foxp3 (1:500; Thermo Fisher Scientific), CD8a (1:500; Thermo Fisher Scientific), or Granzyme B (1:200; Proteintech, Rosemont, IL, USA) for 1 h at 37 °C. Following three washes with PBS, the sections were incubated for 30 min with either Rhodamine (TRITC)-conjugated AffiniPure^®^ Goat Anti-Rat IgG (H + L) or Fluorescein (FITC)-conjugated AffiniPure^®^ Goat Anti-Rabbit IgG (H + L) secondary antibodies (Jackson ImmunoResearch, West Grove, PA, USA). Nuclei were counterstained with DAPI (1:10,000; Invitrogen) for 1 min at room temperature. Whole-slide fluorescence images were acquired using a 3DHISTECH slide scanner (Pannoramic MIDI or similar model). For the quantification, QuPath software (version 0.7.0) was utilized for cell detection and classification.

### Ex vivo cytotoxicity assay

DCs were derived from healthy human peripheral blood mononuclear cells using Ficoll-Hypaque gradient centrifugation (IRB: 2020-06-005BCF#3). Cells were resuspended in AIM V™ Medium (Gibco) at a density of 5 × 10^6^ cells/mL and plated into T75 flasks for a two-hour incubation at 37 °C in 5% CO_2_. Nonadherent cells were gently dislodged by rocking the flasks and then removed. The adherent cells were cultured in AIM V™ Medium supplemented with recombinant human granulocyte–macrophage colony-stimulating factor (1,000 U/mL; R&D Systems, Minneapolis, MN 55413, USA) and interleukin-4 (1,000 U/mL; R&D Systems) to generate DCs. After seven days, the nonadherent cells were harvested by washing, counted, and resuspended in culture medium for further use. For the ex vivo cytotoxicity assay, MDA-MB-231 cells were treated with cobimetinib or DMSO for 24 h, washed with Dulbecco’s phosphate-buffered saline (DPBS), and labeled with CFSE (BD Biosciences) at 37 °C for 15 min. After washing, CFSE-labeled tumor cells were co-incubated with DCs for 4 h. The cells were then washed, resuspended in DPBS, and stained with 7-amino-actinomycin D (7-AAD; BD Biosciences) for flow cytometric analysis. Dead tumor cells were identified as 7-AAD⁺ events within the CFSE⁺ gate.

### Statistical analysis

All statistical analyses were performed using GraphPad Prism 8.0 (GraphPad Software, Inc., San Diego, CA). The results are presented as means ± standard deviation or standard error of the mean from three independent experiments, each performed at least in triplicate. Data analysis was performed using Student’s t-test. Survival curves for mice were generated using the Kaplan-Meier method and compared using the log-rank test. A *P*-value of less than 0.05 was considered statistically significant.

## Results

### Cobimetinib induces cell apoptosis and upregulates DAMPs in murine TNBC cells

**Fig. 1:**
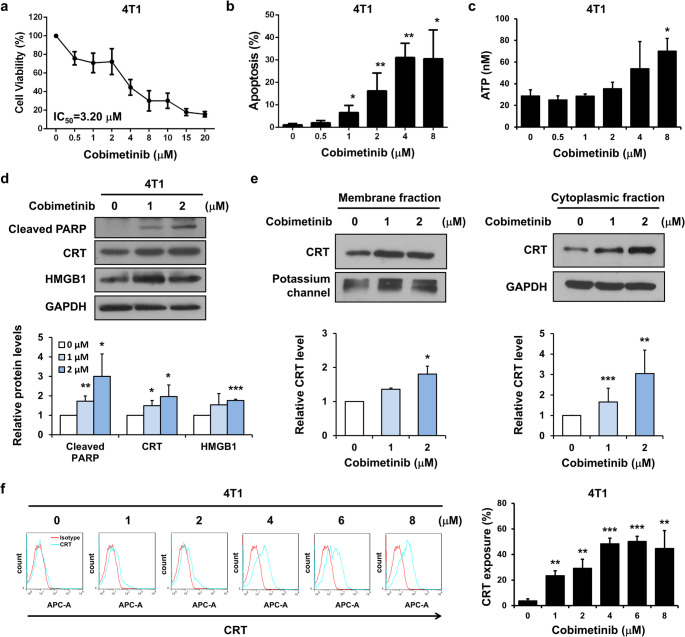
Cobimetinib induces cell apoptosis and upregulates DAMPs in murine TNBC cells. (a–c) 4T1 cells were exposed to varying concentrations of cobimetinib for MTT assays (**a**), flow cytometry analysis (**b**), and ATP measurements (**c**). (**d**) Whole-cell extracts from 4T1 cells treated with cobimetinib or the vehicle were subjected to Western blot analysis. (**e**) Western blot analysis assessed the cytoplasmic and membrane fractions of cells. (**f**) Flow cytometry analysis was employed to examine the surface expression of CRT. Student’s *t*-test, *P < 0.05, **P < 0.01, ***P < 0.001

To evaluate the effects of cobimetinib on DAMPs in TNBC cells, we treated murine 4T1 TNBC cells with cobimetinib and observed a significant suppression of cell viability and induction of cell apoptosis (Fig. 1a and b). Furthermore, cobimetinib treatment elevated extracellular ATP levels and upregulated the expression of CRT and HMGB1 (Fig. 1c and d). Western blot analysis and flow cytometry results also showed increased CRT exposure on the cell membrane (Fig. 1e and f). These findings suggested that cobimetinib induces cell apoptosis and upregulates DAMPs in murine TNBC cells in vitro.

### Cobimetinib elicits CRT expression through ERK inhibition and caspase-8 activation in mouse TNBC cells

**Fig. 2:**
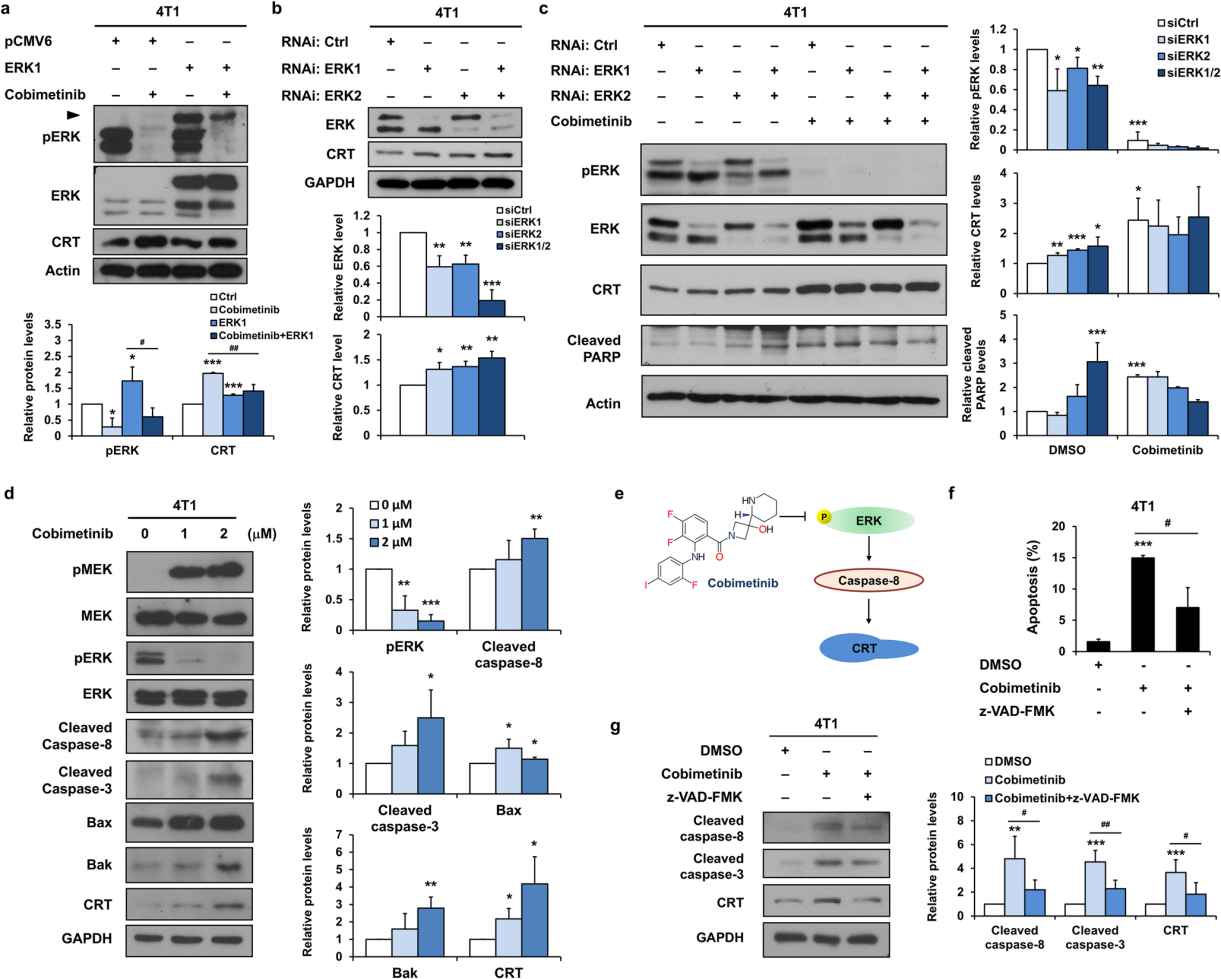
Cobimetinib elicits CRT expression through ERK inhibition and caspase-8 activation. (**a**) 4T1 cells were transfected with plasmids expressing either ERK1 or control for 24 h and subsequently treated with cobimetinib or the vehicle for 18 h, followed by Western blot analysis. The arrow indicates the band of the transfected Myc-DDK-ERK1 (**b**) Western blot analysis of whole-cell extracts from 4T1 cells transfected with siRNA targeting control, ERK1, or ERK2. (**c**) 4T1 cells were transfected with siRNA against control, ERK1, or ERK2 for 24 h and then treated with cobimetinib or the vehicle for 18 h, with subsequent Western blot analysis. (**d**) Western blot analysis of whole-cell extracts from 4T1 cells treated with cobimetinib or the vehicle. (**e**) A pathway diagram showing how cobimetinib induces CRT expression by inhibiting MEK/ERK and activating caspase-8. (**f**) Flow cytometry analysis of apoptotic 4T1 cells treated with cobimetinib for 18 h in the presence or absence of z-VAD-FMK. (**g**) Western blot analysis of whole-cell extracts from 4T1 cells treated with cobimetinib or z-VAD-FMK. Student’s t-test, *P < 0.05, **P < 0.01, ***P < 0.001

To investigate whether cobimetinib induces CRT expression through ERK inhibition, 4T1 cells were transiently transfected with an ERK1 expression construct in the presence or absence of cobimetinib treatment. The results showed that cobimetinib reduced the phosphorylation level of ERK and increased CRT expression, whereas these effects were attenuated by ERK1 overexpression (Fig. 2a). In contrast, knockdown of ERK1 and ERK2 by RNAi slightly increased CRT expression (Fig. 2b). Knockdown of ERK1/2 did not further enhance cobimetinib-induced CRT protein expression or PARP cleavage (Fig. 2c). CRT translocation is evoked by chemotherapy through the PERK-eIF2α pathway, the secretory pathway, and caspase-8 signaling [24]. In addition, phosphorylation of ERK has been reported to inhibit caspase-8-mediated apoptosis in cancer cells [25]. Therefore, we examined whether ERK inhibition by cobimetinib could modulate caspase-8 activation. Cobimetinib treatment induced the cleavage of caspase-8 and caspase-3 and increased the protein expression of Bax and Bak (Fig. 2d). These results suggest that suppression of ERK phosphorylation by cobimetinib activates the caspase-8 apoptotic pathway, which may in turn promote CRT upregulation under cobimetinib treatment (Fig. 2e). The results showed that cobimetinib-induced CRT expression and cell apoptosis were reversed by the caspase inhibitor z-VAD-FMK (Fig. 2f and g), suggesting that cobimetinib elicits CRT expression through ERK inhibition and caspase-8 activation in murine TNBC cells.

### Cobimetinib treatment induces the expression of DAMPs and proapoptotic proteins in human TNBC cells

**Fig. 3:**
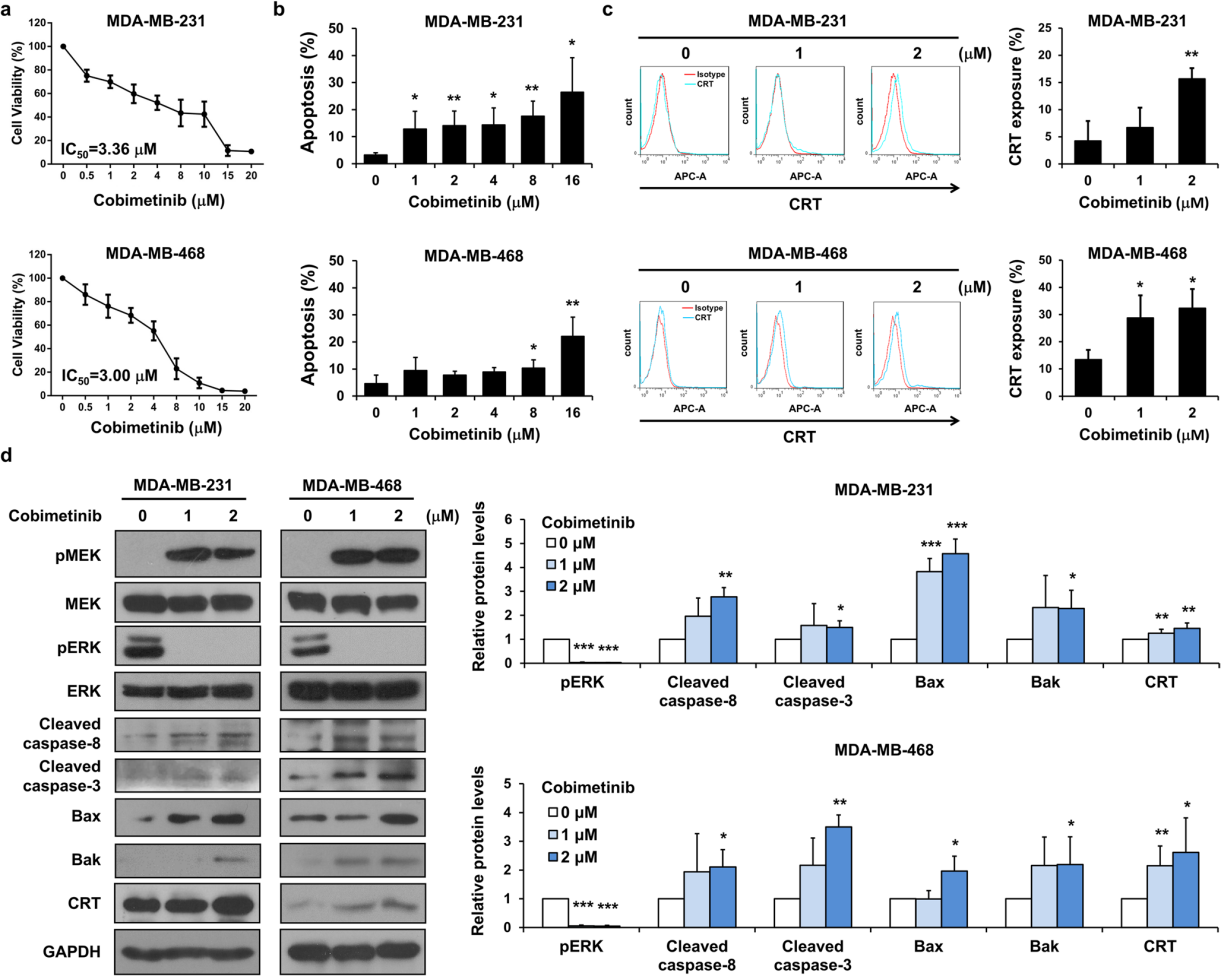
Cobimetinib treatment induces the expression of DAMPs and proapoptotic proteins in human TNBC cells. (**a**) MDA-MB-231 and MDA-MB-468 cells were exposed to cobimetinib at various concentrations for 3 days and evaluated for cell viability through MTT assay. (**b**) Flow cytometry analysis was conducted to assess apoptosis induction in cobimetinib-treated cells. (**c**) Flow cytometry analysis was used to detect surface CRT expression in 4T1 cells treated with cobimetinib or the vehicle. (**d**) Western blot analysis was performed on whole-cell extracts from cobimetinib- or vehicle-treated MDA-MB-231 and MDA-MB-468 cells to examine the expression of pro-apoptotic proteins. Student’s *t*-test, *P < 0.05, **P < 0.01, ***P < 0.001

To validate the effects of cobimetinib on human TNBC cell lines, in vitro studies were conducted using MDA-MB-231 and MDA-MB-468 cells. The results showed that cobimetinib induced apoptosis and promoted CRT exposure on the cell membrane (Fig. 3a-c). Western blot analysis data revealed that cobimetinib treatment activated the caspase-8 axis and increased CRT expression in human TNBC cells (Fig. 3d). These results suggested that cobimetinib treatment induces the expression of DAMPs and proapoptotic proteins in human TNBC cells.

### Cobimetinib suppresses TNBC tumor growth and induces ICD in vivo

**Fig. 4:**
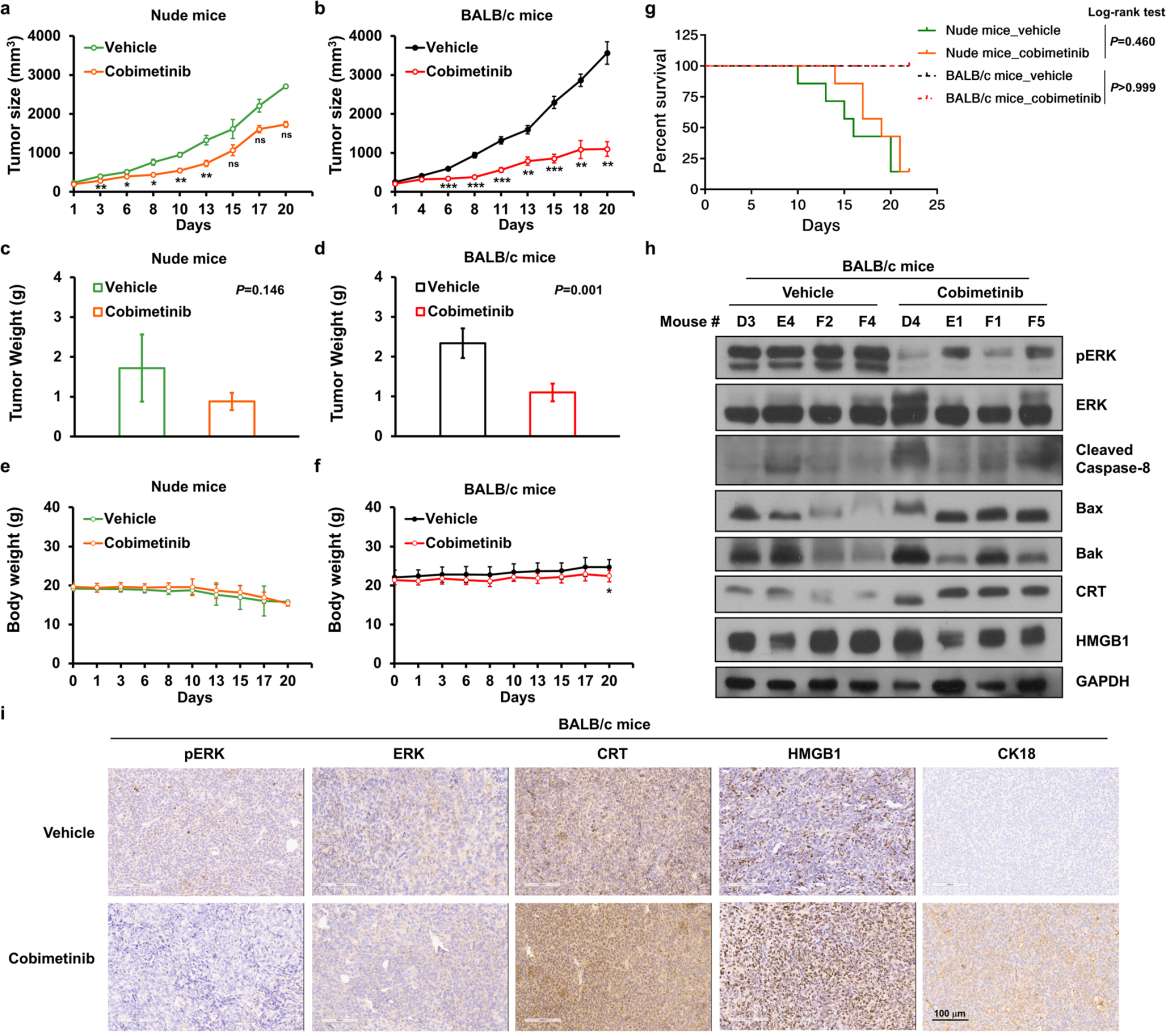
Cobimetinib reduces TNBC tumor growth and induces ICD in vivo. (**a**–**g**) Nude mice and BALB/c mice with 4T1 tumors received treatment with cobimetinib at 10 mg/kg QD or the vehicle. Tumor sizes (**a** and **b**), tumor weights (**c** and **d**), mouse body weights (**e** and **f**), and survival rates (**g**) were measured. (h and i) Xenografted tumors from vehicle-treated mice (designated as D3, E4, F2, and F4) and cobimetinib-treated mice (designated as D4, E1, F1, and F5) were harvested and subjected to Western blot analysis (**h**) and immunohistochemical staining (**i**) using antibodies against pERK, ERK, CRT, HMGB1, and M30 CytoDEATH (CK18). Survival curves represent actual animal survival, not tumor size-based euthanasia; all BALB/c mice remained alive until day 22. Student’s t-test, *P < 0.05, **P < 0.01, ***P < 0.001. Scale bar, 100 μm

According to the operational definition of ICD proposed previously [26], ICD could be demonstrated using in vivo chemotherapy models that compare immunocompetent and immunodeficient mice. We therefore compared the tumor-suppressive effects of cobimetinib in immunodeficient and immunocompetent mice implanted with 4T1 cells. Cobimetinib slightly suppressed tumor growth in nude mice (Fig. 4a and c). In contrast, in BALB/c mice, cobimetinib significantly inhibited tumor growth compared with the vehicle control (Fig. 4b and supplementary Fig. S1a) and markedly reduced tumor weights at the study endpoint (Fig. 4d). The tumor growth inhibition (TGI) values were 37.0% in nude mice and 66.1% in BALB/c mice (Supplementary Fig. 1b), indicating that the antitumor efficacy of cobimetinib was largely immune dependent. Cobimetinib suppressed tumor growth without progressive loss of total body weight. A small but significant difference at day 20 in BALB/c mice (Fig. 4e and f) likely reflects the lower tumor mass in the cobimetinib group, as body weight measurements included the tumor. Moreover, all tumor-bearing BALB/c mice survived throughout the 22-day observation period, whereas both vehicle- and cobimetinib-treated nude mice eventually reached the endpoint. The survival curve of cobimetinib-treated nude mice showed a slightly longer, although not statistically significant, survival compared with the vehicle group (median 19 vs. 16 days; log-rank *P* = 0.460), which is consistent with a direct ERK-inhibitory effect in the absence of T cells (Fig. 4g). In addition, cobimetinib treatment not only inactivated ERK but also activated caspase-8, Bax, Bak, CRT, and HMGB1 in xenografts from BALB/c mice (Fig. 4h and i). The induction of cell apoptosis by cobimetinib treatment was also evidenced by the CK18 apoptotic marker (Fig. 4i). To address local immune responses within the tumor microenvironment, we performed IHC for CD4 and CD8α on tumor tissues. IHC revealed a higher number of intratumoral CD4^+^ and CD8^+^ T cells in cobimetinib-treated tumors relative to vehicle controls (Supplementary Fig. 1c). These results indicated that cobimetinib suppresses TNBC tumor growth and induces DAMPs in vivo.

### Cobimetinib induces antitumor immunity in vivo

**Fig. 5:**
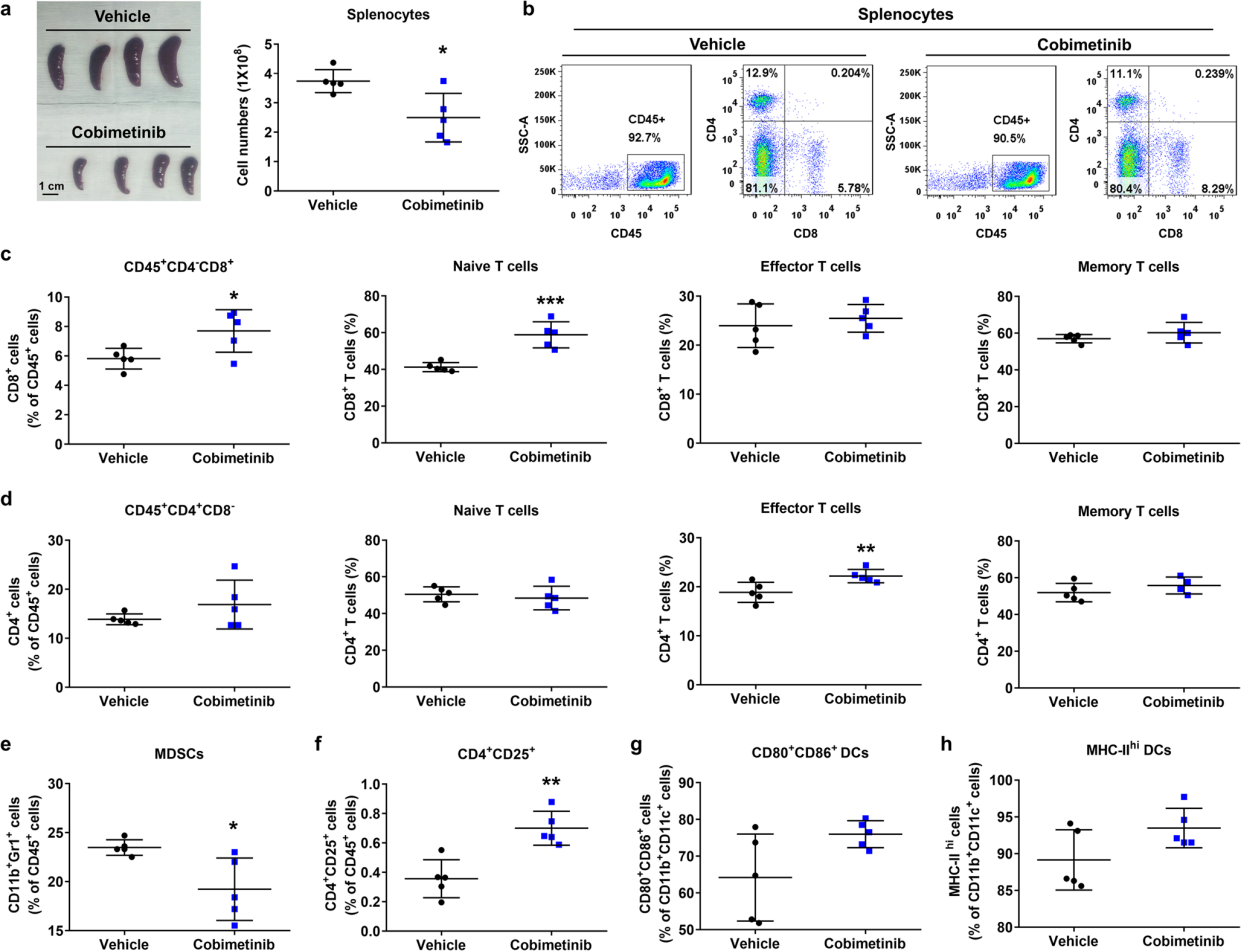
Cobimetinib induces antitumor immunity in vivo. (**a**) Cells were isolated from the spleens of 4T1-bearing BALB/c mice, with a photograph of the spleen (left) and the number of splenocytes (right) shown. (**b**–**h**) Cells were gated on Fixable Viability Stain 780-negative viable cells. CD8 subsets (gated on CD45^+^CD4^−^CD8^+^) include naïve CD8^+^ T cells (CD62L^+^CD44^−^), memory CD8^+^ T cells (CD62L^+^CD44^+^), and effector CD8^+^ T (CD62L^−^CD44^+^) cells. CD4 subsets (gated on CD45^+^CD4^+^CD8^−^) include naïve CD4^+^ T cells (CD62L^+^CD44^−^), memory CD4^+^ T cells (CD62L^+^CD44^+^), and effector CD4^+^ T (CD62L^−^CD44^+^) cells. The percentage of the MDSCs (CD45^+^Gr-1^+^CD11b^+^), CD4^+^CD25^+^ T cells (CD45^+^CD4^+^CD25^+^), mature DCs (CD11b^+^CD11c^+^CD80^+^CD86^+^), and antigen-presenting DCs (CD11b^+^CD11c^+^MHC-II^high^) were quantified. Student’s *t*-test, *P < 0.05, **P < 0.01, *** *P* < 0.001

Induction of ICD results in an effective antitumor immune response by activating DCs and a specific T-cell response [26]. To examine the impact of cobimetinib on the immune response in vivo, we examined the populations of immune cells. 4T1 tumor-bearing BALB/c mice were administered cobimetinib or vehicle for 22 days. Tumor-bearing mice receiving vehicle had larger spleens than those receiving cobimetinib but no obvious spleen metastasis (Fig. 5a and supplementary Fig. 2a). Fresh cells from the spleens of mice were isolated for flow cytometry analysis (Fig. 5b). The data showed that cobimetinib treatment increased the number of CD8^+^ T cells in the spleen of 4T1 tumor-bearing mice. The levels of naïve CD8^+^ T cells and effector CD4^+^ T cells were increased with cobimetinib treatment (Fig. 5c and d). Cobimetinib treatment markedly suppressed myeloid-derived suppressor cells (MDSCs; Fig. 5e). Cobimetinib led to an increase in the proportion of CD4^+^CD25^+^ T cells in the spleen (Fig. 5f). However, further analysis of FOXP3 expression showed that the CD4^+^CD25^+^FOXP3^+^ population did not increase, but instead exhibited a decreasing trend without statistical significance (Supplementary Fig. 2b). These findings suggest that the expanded CD4^+^CD25^+^ population likely includes FOXP3-negative subsets, consistent with activated effector CD4^+^ T cells, rather than an expansion of regulatory T cells. Furthermore, cobimetinib exhibited a tendency toward elevated proportions of CD80^+^CD86^+^ (mature) and MHC-II^high^ (antigen-presenting) DCs (Fig. 5g and h). To elucidate the functional states of TILs in 4T1 tumors, we performed immunofluorescence staining. Cobimetinib treatment significantly reduced the proportion of Foxp3^+^ regulatory T cells within the CD4^+^ TIL population compared to vehicle control (Supplementary Fig. 2c). Furthermore, we observed that CD8^+^ T cells in both groups predominantly co-expressed Granzyme B, with a significantly higher abundance of these CD8^+^Granzyme B^+^ cells in the cobimetinib-treated tumors (Supplementary Fig. 2d). To further evaluate whether cobimetinib-induced tumor cell death can stimulate immune effector function, we performed a human ex vivo CFSE/7-AAD cytotoxicity assay. Flow cytometric analysis showed an increased proportion of 7-AAD^+^ (dead) tumor cells after co-culture with DCs primed by cobimetinib-treated tumor cells (Supplementary Fig. 3), suggesting that cobimetinib-induced ICD enhances DC-mediated cytotoxicity.

### Cobimetinib alters immune cell populations in non–tumor-bearing mice

**Fig. 6:**
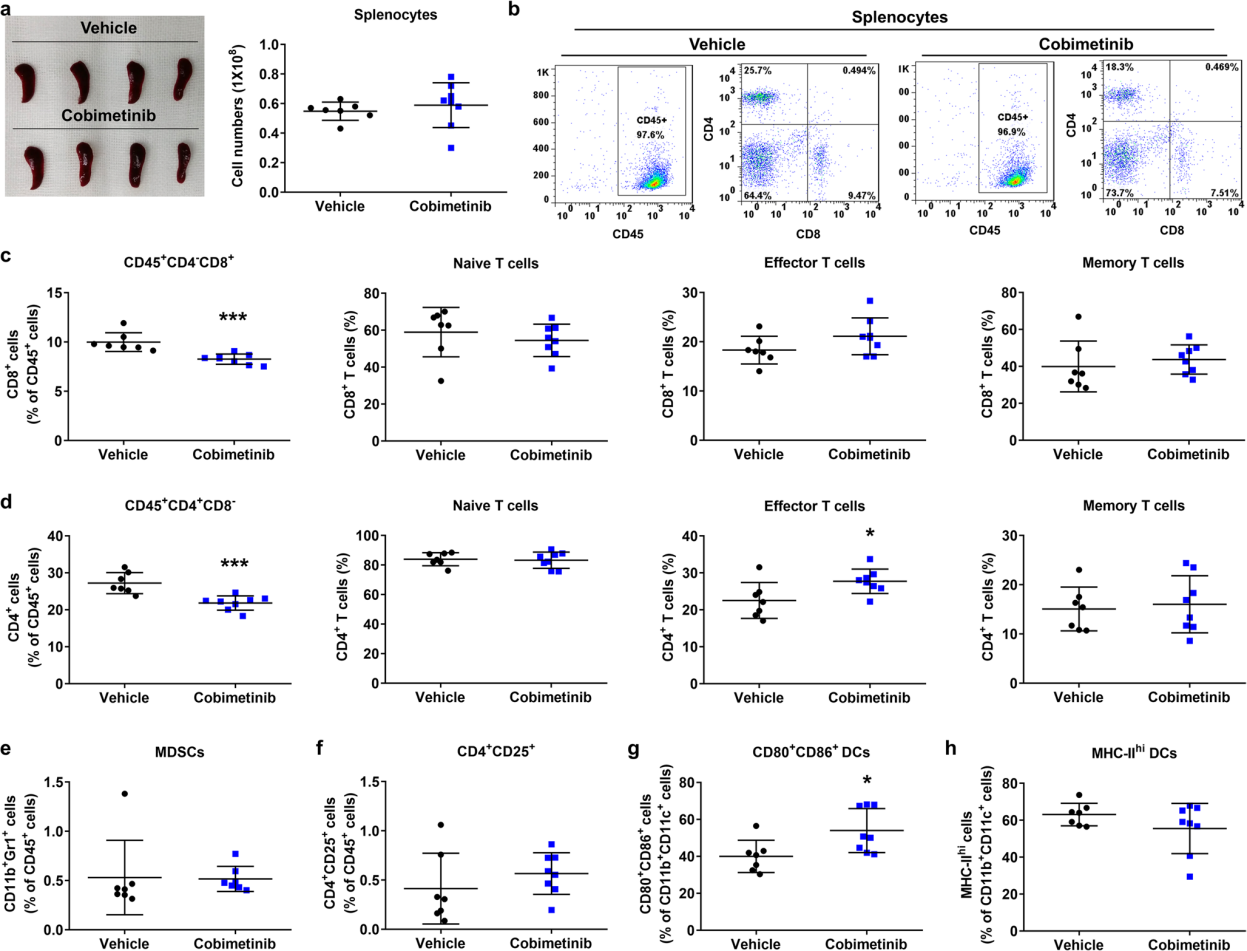
The effects of cobimetinib treatment on non–tumor-bearing mice. (**a**) Non–tumor-bearing BALB/c mice were treated with cobimetinib at 10 mg/kg QD or vehicle. A photograph of the spleen (left) and the number of splenocytes (right) are shown. (**b**–**h**) Fresh cells were isolated from the spleens of non–tumor-bearing BALB/c mice for flow cytometry analysis. The subpopulations of CD4+ and CD8+ T cells, MDSCs, CD4+CD25+ T cells, CD80+CD86+ DCs, and MHC-II^high^ DCs were quantified. Student’s t-test, *P < 0.05, ***P < 0.001

To further explore whether the immunomodulatory effects of cobimetinib depend on the presence of tumors, we examined immune-cell profiles in non–tumor-bearing BALB/c mice treated with cobimetinib or vehicle. There were no differences in spleen size or body weight between vehicle- and cobimetinib-treated mice (Fig. 6a and Supplementary Fig. 4a). Interestingly, cobimetinib treatment increased the numbers of effector CD4^+^ T cells and CD80^+^CD86^+^ DCs, while reducing the numbers of total CD8^+^ and CD4^+^ T cells in the spleens of non–tumor-bearing mice (Fig. 6b–h and Supplementary Fig. 4b). However, increases in the proportions of total CD8^+^ T cells, naïve CD8^+^ T cells, and CD4^+^CD25^+^ T cells, as well as a decrease in the proportion of MDSCs, were observed only in tumor-bearing mice receiving cobimetinib (Supplementary Table 1). Consistently, the increase in CD4^+^CD25^+^ T cells was evident in cobimetinib-treated spleens of tumor-bearing mice (Fig. 5f) but not in non–tumor-bearing mice (Fig. 6f), suggesting that this population likely reflects activation of CD4^+^ T cells in response to tumor challenge. These findings indicated that cobimetinib did not trigger overt immune activation or systemic toxicity in the absence of tumors, but exerted mild immunomodulatory effects distinct from the ICD-associated immune activation observed in tumor-bearing mice.

## Discussion

In this study, we found that cobimetinib treatment not only suppressed tumor growth as a MEK inhibitor but also elicited immune responses consistent with ICD in vivo. Immunocompetent mice exhibited a more favorable response to cobimetinib than immunodeficient mice, supporting an immune-dependent antitumor effect. Cobimetinib treatment increased the total number of splenic CD8⁺ T cells in tumor-bearing mice but not in non-tumor-bearing mice. Moreover, the basal proportion of MDSCs in tumor-bearing mice was higher than that in non–tumor-bearing mice, and cobimetinib administration significantly decreased the MDSC population. Mechanistically, cobimetinib inhibited ERK phosphorylation and induced the expression of DAMPs, including increased CRT exposure. In addition, cobimetinib-treated tumor cells enhanced DC-mediated cytotoxicity, further supporting ICD-associated immune activation. These immunomodulatory effects are consistent with the known consequences of ICD, which can promote antigen presentation and the activation of effector T cells. In the absence of functional T cells, as in nude mice, cobimetinib treatment resulted in only transient tumor control despite similar direct inhibition of MEK/ERK signaling. Therefore, the greater tumor suppression observed in immunocompetent (BALB/c) mice likely reflects both the direct antiproliferative effects of MEK inhibition and the indirect, immune-mediated antitumor mechanisms. The immunophenotyping data from non–tumor-bearing mice indicate that cobimetinib treatment alone did not induce strong immune activation, supporting its favorable tolerability profile. These results contrast with the more pronounced immune engagement observed in tumor-bearing mice, suggesting that cobimetinib’s immune-stimulatory effects are context dependent and influenced by tumor-associated factors. Collectively, these findings suggest that cobimetinib exerts immune-dependent antitumor activity in TNBC.

MEK inhibitors such as selumetinib have been reported to induce apoptosis by increasing cleaved caspase levels [27]. Previous studies have suggested that inducing CRT exposure is a potential approach for immunogenic chemotherapy [28]. In our study, we found that cobimetinib treatment activated caspase-8 signaling, leading to the increased expression and membrane localization of CRT. Caspase-8, Bax, and Bak activation have been shown to be responsible for CRT exposure in ICD [29], and our data confirmed the role of caspase-8 in cobimetinib-induced CRT exposure. However, CRT exposure can also be induced by caspase-8-independent mechanisms [30]. For instance, treatment with a caspase-8 inhibitor does not affect the induction of CRT by photodynamic therapy utilizing the photosensitizer hypericin [30].

Mounting evidence suggests that MEK inhibition can modify the tumor-immune microenvironment through various mechanisms. For instance, trametinib-mediated MEK inhibition has been demonstrated to increase the expression of MHC-1, thereby enhancing the immunogenicity of breast cancer [31]. Furthermore, trametinib has the ability to inhibit the expansion of MDSCs driven by osteopontin and suppress the growth of primary mammary tumor models driven by KRAS [32]. MEK inhibition, either alone or in combination with antibodies targeting the immune checkpoint proteins PD-1 and PD-L1, has also been shown to increase the levels of TILs and improve immunotherapy outcomes in animal cancer models [18, 33–35]. Limagne et al. observed that MEK inhibitors including selumetinib, cobimetinib, and trametinib in combination with pemetrexed and cisplatin (PEM/CDDP, standard-of-care chemotherapy for non-small cell lung cancer) enhanced PEM/CDDP-induced CXCL10 expression. Mechanistically, they demonstrated that PEM/CDDP plus a MEK inhibitor (mainly trametinib) promotes optineurin-dependent mitophagy, resulting in CXCL10 production in a mitochondrial DNA- and TLR9-dependent manner [36]. However, several studies indicate paradoxical conclusions regarding the immunomodulatory effects of MEK inhibitors. The impact of MEK inhibition on T-cell function may depend on the specific context [37]. Furthermore, the majority of MEK inhibitors, such as cobimetinib and selumetinib, do not disrupt the phosphorylation of the activation loop sites on MEK1/2. This results in the accumulation of phosphorylated MEK1/2, which differs from the effect of trametinib [38]. It is noteworthy that the ICD-inducing effect of cobimetinib observed in our study appears to be independent of KRAS mutation status. All TNBC models used were KRAS wild-type, yet cobimetinib effectively induced ICD and downstream immune activation. Previous studies have similarly shown that MEK inhibitors such as trametinib and selumetinib exert pro-apoptotic and immunomodulatory effects across multiple tumor types irrespective of KRAS mutation [37].

Induction of ICD is considered a novel strategy for cancer treatment. ER stress and ROS production are crucial for releasing DAMPs, which are elicited by dying cancer cells and then activate DC and T-cell responses. Recently, several bona fide ICD inducers, such as doxorubicin and cyclophosphamide, have been identified that induce danger signals, thereby exerting immune responses [39]. Currently, immune induction by chemotherapeutics is being assessed in clinical trials [40]. In the TONIC trial, patients with metastatic TNBC received the PD-1 inhibitor nivolumab alone or in combination with irradiation, cyclophosphamide, cisplatin, or doxorubicin. The overall response rate in the total cohort was 20%, whereas induction with doxorubicin prior to nivolumab achieved a response rate of 35%. Doxorubicin pre-treatment was shown to induce inflammatory phenotypes and enhance the efficacy of PD-1 blockade [41]. These clinical observations support the concept that therapeutic regimens capable of inducing ICD can potentiate the response to ICIs. Although this study focused on TNBC because of its immunogenic nature and high MAPK pathway activity, the ability of MEK inhibition to trigger ICD and modulate immune responses may extend to other cancer types. Further investigation into whether similar immune-stimulatory effects of cobimetinib occur in other solid tumors would be of considerable interest.

While our immune profiling in the spleen indicates systemic immune modulation associated with cobimetinib, we acknowledge that immunophenotyping of the bone marrow and tumor tissue by flow cytometry and immunostaining was not performed in this study. In particular, profiling the tumor-draining lymph nodes and tumor-resident immune populations could reveal changes in antigen-presenting cell maturation, as well as the proportions of exhausted and effector-like T cells, providing a more comprehensive view of local antitumor immunity. Functional characterization of cytokine and cytotoxic markers such as IFNγ, TNFα, granzyme B, perforin, and CD107a would further clarify the immune contexture of cobimetinib-treated tumors. Moreover, using an antigen-defined system, such as a C57BL/6-background breast tumor line (H-2Kᵇ) expressing OVA together with OT-I T cells [42], could directly link cobimetinib-induced antigen release to tumor-specific T-cell responses. Such analyses would provide additional support for enhanced local immune activation and more direct evidence of ICD-associated antitumor immunity. The absence of these analyses represents a limitation of the current study.

In summary, our findings demonstrate that cobimetinib acts as an ICD inducer by promoting CRT expression through the activation of the caspase-8 signaling pathway. Cobimetinib exerts antitumor activity and immunomodulatory effects in TNBC.

## Electronic Supplementary Material

Below is the link to the electronic supplementary material.

Supplementary Material 1

Supplementary Material 2

## Data Availability

The data that supports the findings of this study are available in the supplementary material of this article**.**
