# Supplementary figures and images for: MEK inhibitor cobimetinib increases calreticulin and induces immune modulation in TNBC
Source: J Mol Med (Berl). 2026 May 25;104(1):80. doi: 10.1007/s00109-026-02684-8 (PMC13201367; doi:10.1007/s00109-026-02684-8)

**Supporting information**


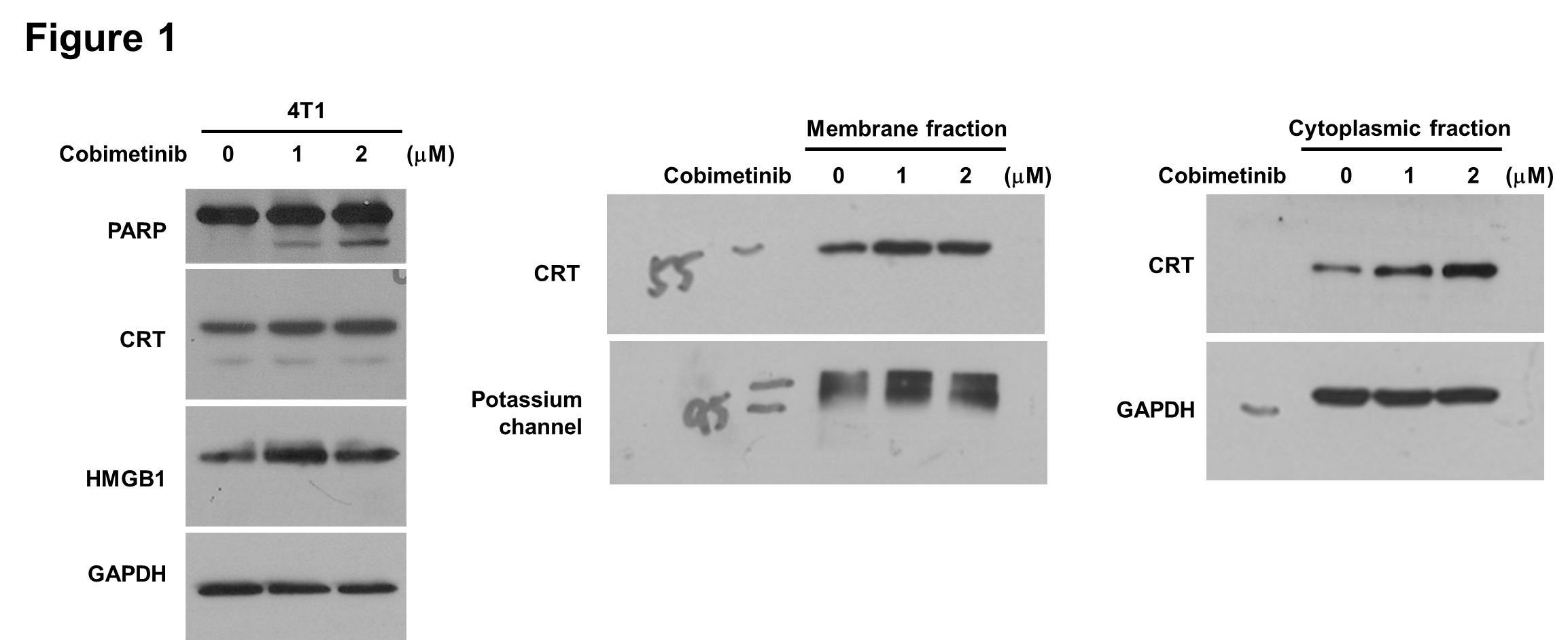


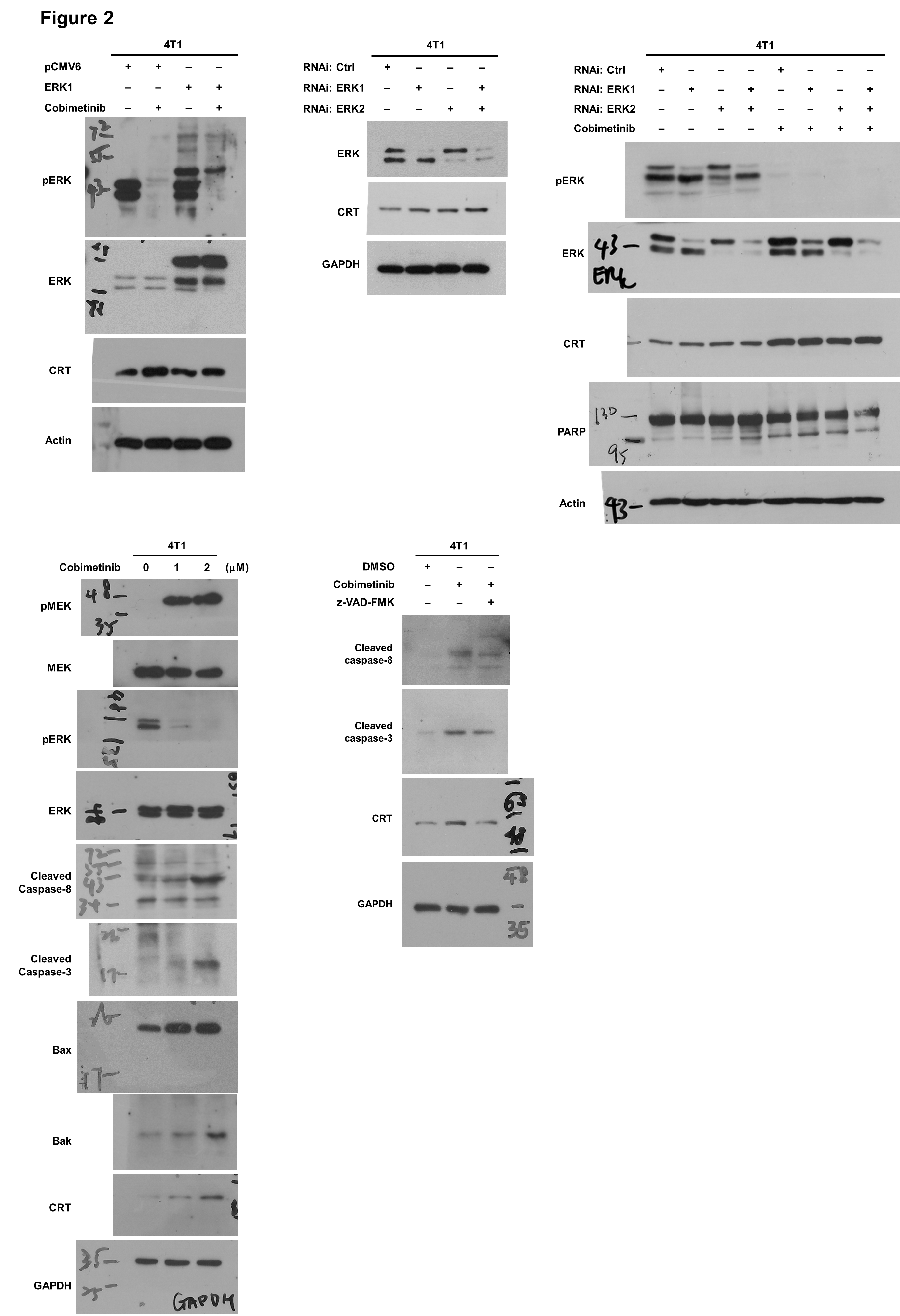


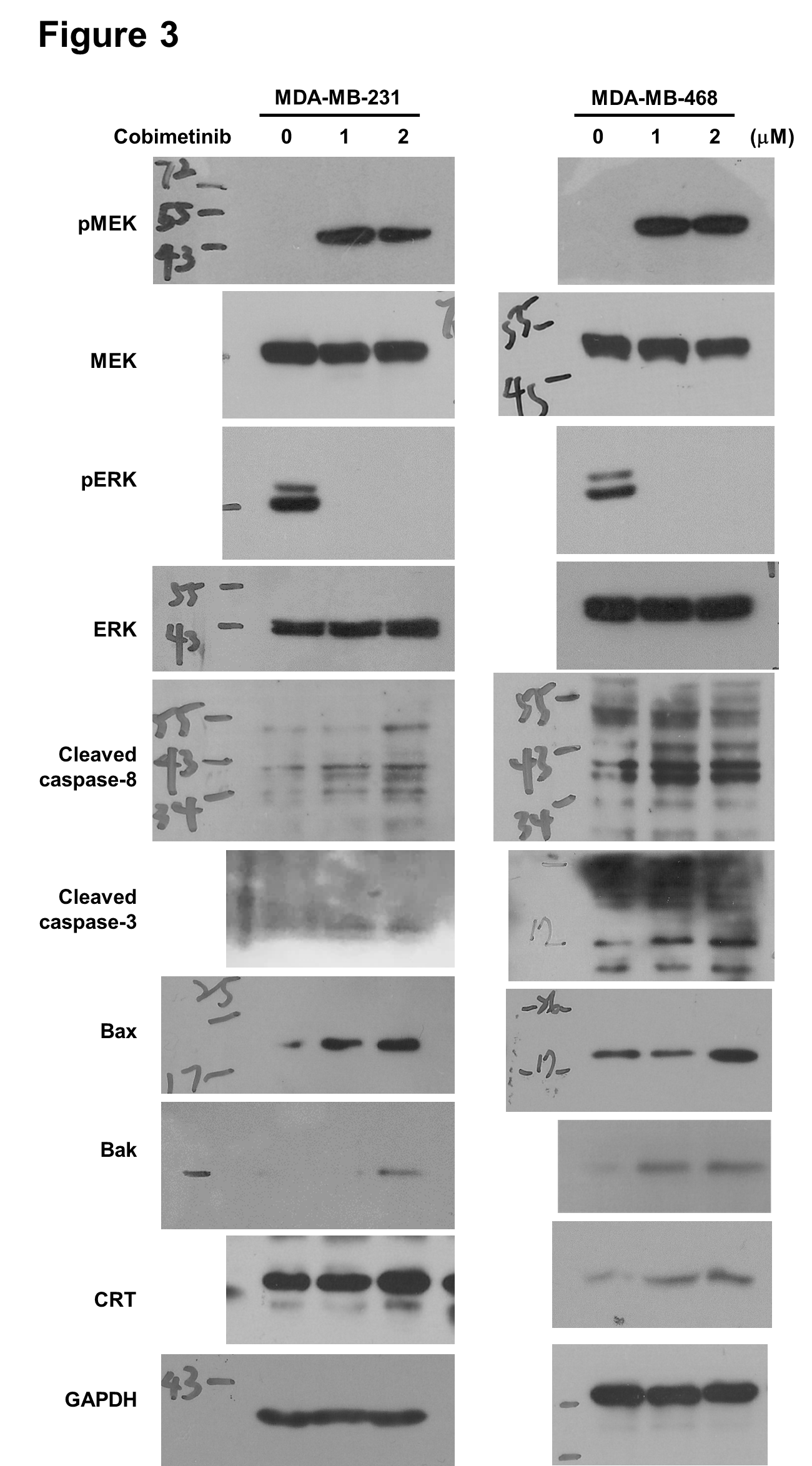


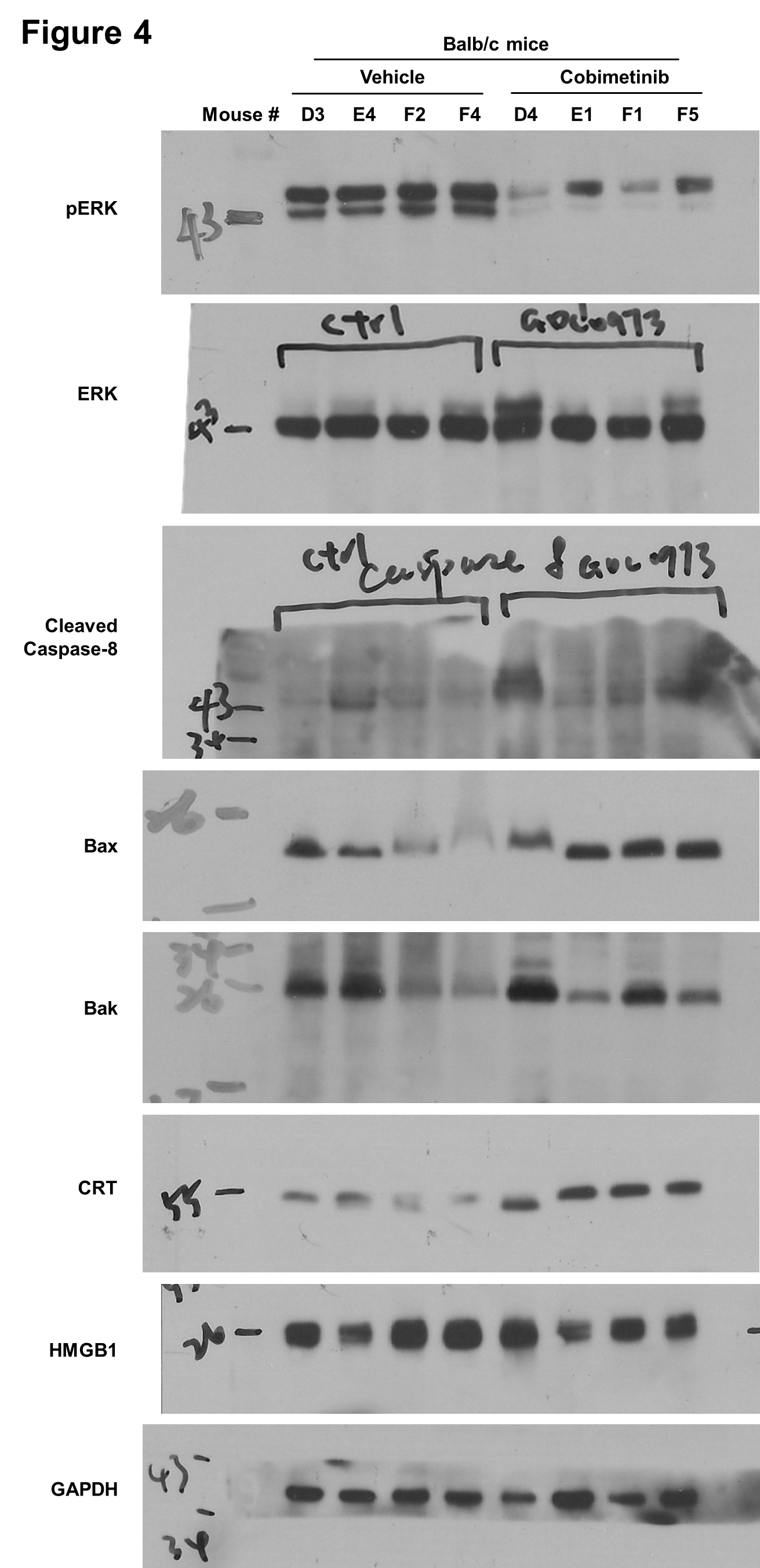

Supplement: Supplementary file 1 — Supplementary Material 1 [file 109_2026_2684_MOESM1_ESM.docx]
