# Supplementary material for: MEK inhibitor cobimetinib increases calreticulin and induces immune modulation in TNBC
Source: J Mol Med (Berl). 2026 May 25;104(1):80. doi: 10.1007/s00109-026-02684-8 (PMC13201367; doi:10.1007/s00109-026-02684-8)
Supplement: Supplementary file 2 — Supplementary Material 2 [file 109_2026_2684_MOESM2_ESM.docx]

**Supporting information**

**Table S1. The cell populations of tumor-bearing mice or non–tumor-bearing mice that received cobimetinib or vehicle.**

| **Cell populations** | **Tumor-bearing mice** | | ***P*-value** | **Non–tumor-bearing mice** | | ***P*-value** |
| --- | --- | --- | --- | --- | --- | --- |
|  | **Vehicle** | **Cobimetinib** |  | **Vehicle** | **Cobimetinib** |  |
| **Total CD4 T cells (% in CD45^+^ gate)** | 13.86±1.10 | 16.88±4.98 | 0.222 | 27.2±2.84 | 21.83±1.93 | 0.001 |
| **Naïve CD4 T cells (% in CD45^+^CD4^+^ gate)** | 49.85±4.42 | 50.2±5.87 | 0.927 | 83.86±4.41 | 83.23±5.49 | 0.811 |
| **Memory CD4 T cells (% in CD45^+^CD4^+^ gate)** | 53.13±4.79 | 56.28±4.98 | 0.397 | 15.06±4.46 | 16.01±5.79 | 0.729 |
| **Effector CD4 T cells (% in CD45^+^CD4^+^ gate)** | 18.20±1.63 | 21.63±0.63 | 0.008 | 22.51±4.88 | 27.71±3.30 | 0.029 |
| **Total CD8 T cells (% in CD45^+^ gate)** | 5.81±0.70 | 7.69±1.45 | 0.031 | 9.98±0.95 | 8.25±0.52 | 0.001 |
| **Naïve CD8 T cells (% in CD45^+^CD8^+^ gate)** | 41.22±2.45 | 58.80±7.07 | 0.001 | 58.93±13.36 | 54.46±8.77 | 0.452 |
| **Memory CD8 T cells (% in CD45^+^CD8^+^ gate)** | 56.96±2.29 | 60.22±5.59 | 0.262 | 39.91±13.78 | 43.71±7.92 | 0.517 |
| **Effector CD8 T cells (% in CD45^+^CD8^+^ gate)** | 23.96±4.46 | 25.46±2.82 | 0.543 | 18.30±2.81 | 21.10±3.75 | 0.130 |
| **MDSCs (% in CD45^+^ gate)** | 23.48±0.79 | 19.22±3.19 | 0.020 | 0.53±0.38 | 0.52±0.13 | 0.929 |
| **CD4^+^CD25^+^ Tregs (% in CD45^+^ gate)** | 0.36±0.13 | 0.70±0.12 | 0.002 | 0.41±0.36 | 0.57±0.21 | 0.325 |
| **CD80^+^CD86^+^ DCs (% in CD45^+^CD11b^+^CD11c^+^ gate)** | 64.18±11.85 | 75.98±3.67 | 0.066 | 39.97±8.71 | 53.94±11.92 | 0.024 |
| **MHC-II^high^ DCs (% in CD45^+^CD11b^+^CD11c^+^ gate)** | 89.14±4.10 | 93.48±2.68 | 0.083 | 63.07±6.10 | 55.50±13.61 | 0.199 |

Splenocytes from BALB/c mice with 4T1 tumors were treated with either cobimetinib or vehicle, and splenocytes from nontumor-bearing mice (healthy BALB/c mice) were treated with cobimetinib or vehicle. These samples underwent flow cytometry analysis, with a total of 10,000 events collected for T cell analysis, and 35,000 events each collected for MDSCs and DCs analysis.


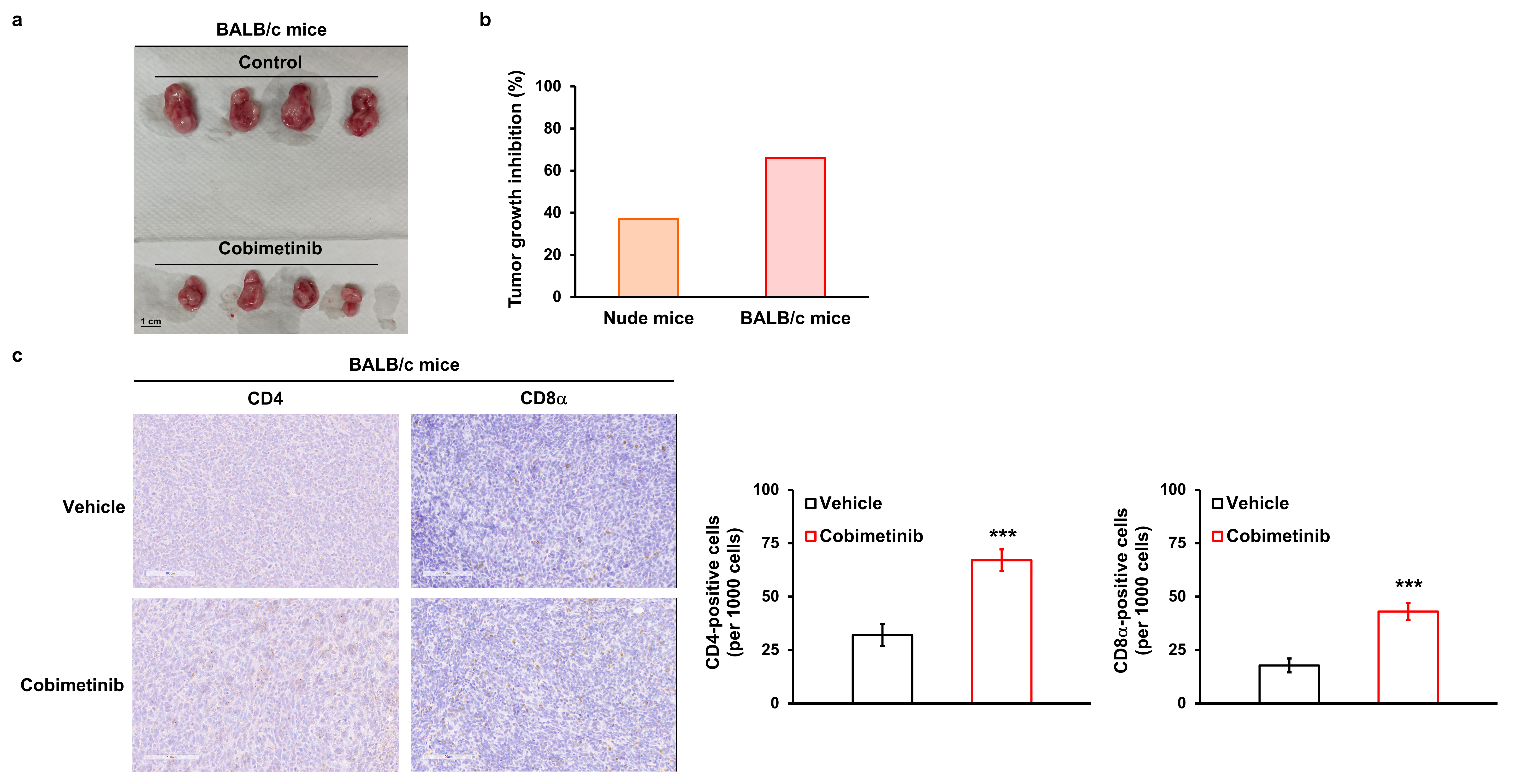


**Figure S1. Cobimetinib treatment suppresses tumor growth**

(a) Related to Figure 4. Representative photograph of 4T1 xenografts excised from BALB/c mice treated with vehicle or cobimetinib. (b) Tumor growth inhibition (TGI) rates in nude and BALB/c mice. TGI was calculated as [1 − (ΔCobimetinib / ΔVehicle)] × 100, where Δ represents the mean change in tumor volume from day 0 to the endpoint for each group. Error bars are not shown because TGI values were derived relative to the control group. (c) Representative immunohistochemical staining for CD4 and CD8α in tumor sections from BALB/c mice treated with vehicle or cobimetinib (left). Quantification of CD4- or CD8α-positive cells per 1000 tumor cells is shown (right). Student’s t-test, ****P* < 0.001.


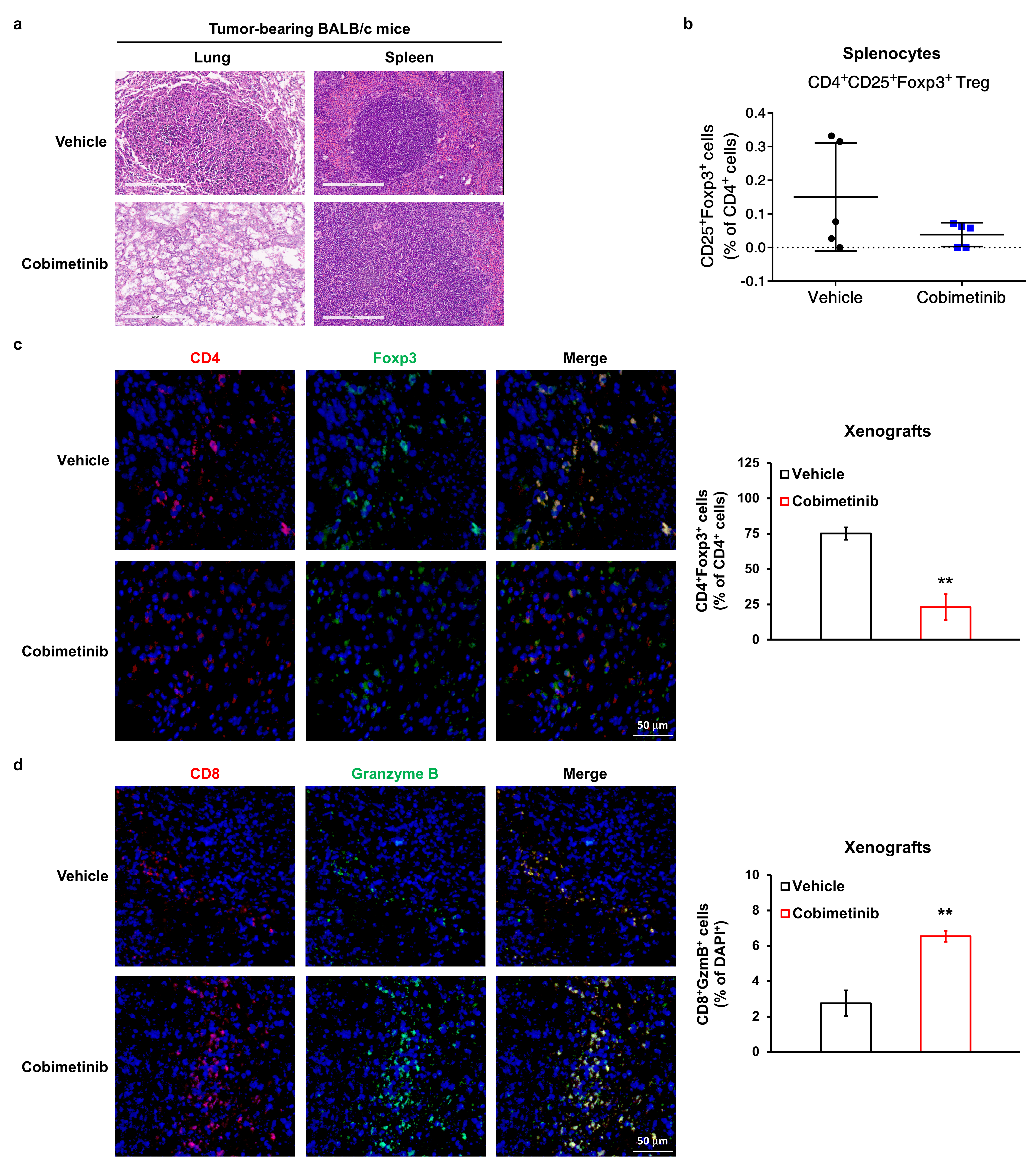


**Figure S2. The effects of cobimetinib treatment on 4T1 tumor-bearing BALB/c mice**

(a) H&E staining of lung and spleen tissues from 4T1 tumor-bearing mice. (b) Flow cytometric analysis of splenic regulatory T cells (Tregs). The percentage of CD25^+^Foxp3^+^ Tregs (within the CD45^+^CD4^+^ population) in splenocytes is shown. (c) Representative immunofluorescence images of CD4 (red) and Foxp3 (green) in tumor sections from BALB/c mice treated with vehicle or cobimetinib (left). Quantification of the proportion of CD4^+^Foxp3^+^ Tregs within the CD4^+^ population in the tumor is shown (right). (d) Representative immunofluorescence images of CD8 (red) and Granzyme B (GzmB, green) in tumor sections (left). Quantification of the frequency of CD8^+^GzmB^+^ cells relative to total DAPI^+^ nuclei in the tumor is shown (right). Nuclei were counterstained with DAPI (blue). Merged images display overlaid signals from all channels. Scale bars, 50 μm. Data are presented as mean ± SD. Student’s t-test, ***P* < 0.01.


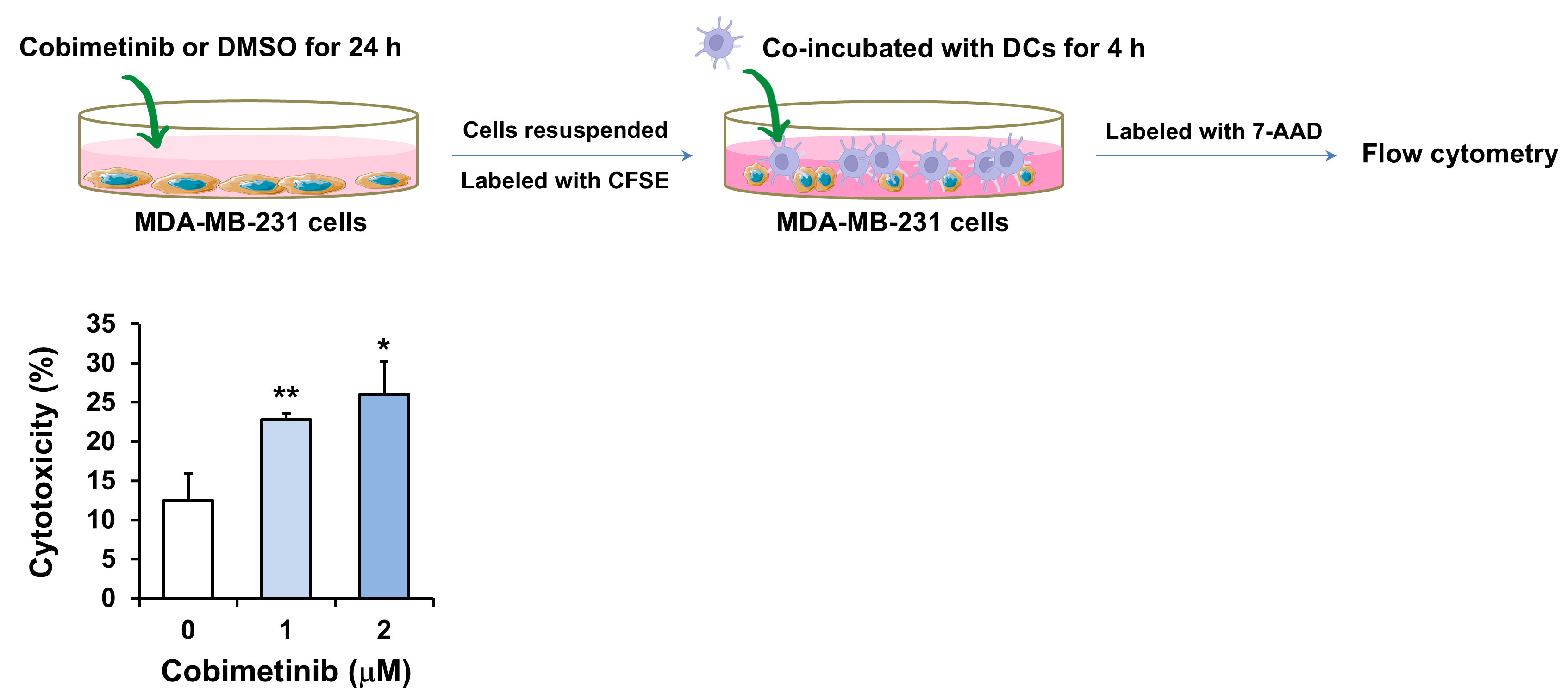


**Figure S3. Cobimetinib-induced tumor cell death enhances dendritic cells-mediated cytotoxicity.**

MDA-MB-231 cells were treated with cobimetinib or DMSO for 24 h, harvested and labeled with CFSE, and then co-incubated with healthy human dendritic cells (DCs) for 4 h. After co-culture, cells were labeled with 7-AAD and analyzed by flow cytometry to determine tumor-cell death. Student’s t-test, **P* < 0.05, ***P* < 0.01.


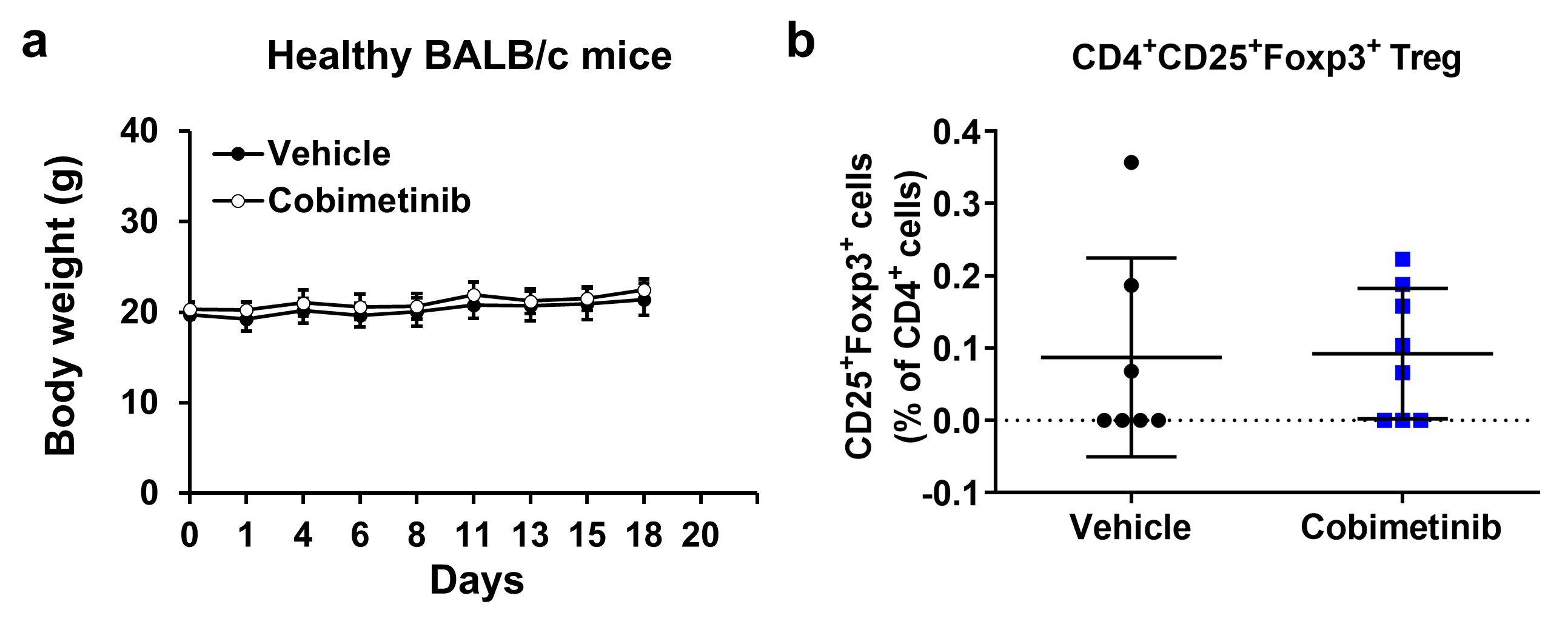


**Figure S4.** **The effects of cobimetinib treatment on non–tumor-bearing mice.**

(a) Related to Figure 6. BALB/c mice were treated with cobimetinib at 10 mg/kg QD or vehicle and the body weight of mice were evaluated as the figure shown. (b) Flow cytometric analysis of splenic regulatory T cells (Tregs). The percentage of CD25^+^Foxp3^+^ Tregs (within the CD45^+^CD4^+^ population) in splenocytes is shown.
